# Characterization of a Low Shrinkage Dental Composite Containing Bismethylene Spiroorthocarbonate Expanding Monomer

**DOI:** 10.3390/ijms15022400

**Published:** 2014-02-10

**Authors:** Jing Fu, Wenjia Liu, Zhichao Hao, Xiangnan Wu, Jian Yin, Anil Panjiyar, Xiaoqing Liu, Jiefei Shen, Hang Wang

**Affiliations:** 1 Department of Prosthodontics, Affiliated Hospital of Medical College, Qingdao University, Qingdao 266003, Shandong, China; E-Mail: quietfly@163.com; 2 State Key Laboratory of Oral Diseases, West China Hospital of Stomatology, Sichuan University, Chengdu 610041, Sichuan, China; E-Mails: simmer0728@sina.cn (W.L.); haozi0548@163.com (Z.H.); xiangnanwu01@gmail.com (X.W.); yinjian1000@126.com (J.Y.); dranilPanjiyar@gmail.com (A.P.); shenjiefei@scu.edu.cn (J.S.); 3 Department of Dental Materials, West China Collage of Stomatology, Sichuan University, Chengdu 610041, Sichuan, China; E-Mail: xqliu5102@sina.com

**Keywords:** unsaturated bismethylene spiroorthocarbonate, dental composites, compressive strength, volumetric shrinkage, contraction stress

## Abstract

In this study, a novel dental composite based on the unsaturated bismethylene spiroorthocarbonate expanding monomer 3,9-dimethylene-1,3,5,7-tetraoxa-spiro[5,5]undecane (BMSOC) and bisphenol-*S*-bis(3-meth acrylate-2-hydroxypropyl)ether (BisS-GMA) was prepared. CQ (camphorquinone) of 1 wt % and DMAEMA (2-(dimethylamino)ethyl methacrylate) of 2 wt % were used in a photoinitiation system to initiate the copolymerization of the matrix resins. Distilled water contact angle measurements were performed for the wettability measurement. Degree of conversion, volumetric shrinkage, contraction stress and compressive strength were measured using Fourier Transformation Infrared-FTIR spectroscopy, the AccuVol and a universal testing machine, respectively. Within the limitations of this study, it can be concluded that the resin composites modified by bismethylene spiroorthocarbonate and BisS-GMA showed a low volumetric shrinkage at 1.25% and a higher contact angle. The lower contraction stress, higher degree of conversion and compressive strength of the novel dental composites were also observed.

## 1. Introduction

Light-curing resin composites have been used more and more widely for restorative dentistry. These materials not only have superior physiochemical performance and manipulative qualities, but also acceptable appearance and favorable biocompatibility. They have the advantage of directly bonding to the tooth structure without the sacrifice of removing healthy tissues. As a result, light-curing resin composites are an area of research interest in many aspects [1–4].

The age of modern dental composites starts with Bowen’s resin, 2,2-bis[4(2-hydroxy-3-methacryloylpropyloxy)phenyl]propane (Bis-GMA), which is the primary organic ingredient in nearly every commercial restorative resin because of the outstanding ability to form cross-links (which are stronger than linear polymers) during polymerization. Although the composite based on Bis-GMA has become vital for dental restoration due to its superior aesthetic quality, simple administration, and enhanced mechanical strength, there are still problems. An important issue of the monomer phase is that it undergoes polymerization shrinkage. Polymerization shrinkage [5] and subsequent contraction stresses [6] can lead to post-operative sensitivity, marginal discoloration, secondary caries, cuspal displacement and even cracks in healthy tooth structure [7,8]. Therefore, eliminating or reducing the amount of volumetric contraction during polymerization is one of the most important issues in the development of dental composites.

The volumetric shrinkage of resin composites during polymerization arises from two different factors: the van der Waals distance between the monomer molecules is replaced by a covalent bond during polymerization, and the intermolecular distance between the polymer chains becomes smaller than that between the monomers. Numerous studies aimed at developing resin composites with a low level of curing shrinkage have been performed [9–11]. Many efforts have been directed toward modifying Bis-GMA, such as substitution of a hydrogen atom, methyl or trimethicone for the hydroxyl group [12], and so on. Since a double ring-opening polymerization was reported in 1972 [13], various monomers based on spiroorthoester (SOE) [14,15], bicycloorthoester (BOE) [16], and spiroorthocarbonate (SOC) [17–19] have been developed that indeed exhibit volumetric expansion upon polymerization. Among these, spiroorthocarbonates (SOCs) have been the most widely investigated because they show the most significant volume expansions due to the compact nature of the bicyclic monomer and the relatively more flexible open-chain structure associated with the polymer.

A goal of our long-time work has been to develop monomers that can mitigate polymerization stress and shrinkage in dental resin systems. In our previous study, BisS-GMA (bisphenol-*S*-bis (3-methacrylato-2-hydroxy propyl) ether) was synthetized [20]. In comparison to Bis-GMA, BisS-GMA exhibited lower volumetric shrinkage and better polymerization activities because of the strongly polar sulfonyl (O=S=O). The specific aim of this investigation is to develop a novel dental photocurable resin composite based on BisS-GMA as a resin matrix monomer and the expanding monomer unsaturated spiroorthocarbonates3,9-dimethylene-1,3,5,7-tetraoxa-spiro[5,5]undecane (BMSOC), mixed with the diluent striethylene glycol dimethacrylate(TEGDMA) at a certain ratio. CQ (camphorquinone)/DMAEMA (2-(dimethylamino) ethyl methacrylate) were used as the photoinitiation system to initiate the copolymarization of Bis-GMA/BisS-GMA with unsaturated BMSOC expanding monomer. Then, we evaluated and compared the wettability, degree of conversion, volumetric shrinkage, contraction stress and compressive strength of the resin composites.

## 2. Results and Discussion

### 2.1. Copolymerization of the Matrix Resins and Degree of Conversion

**Figure 1.**
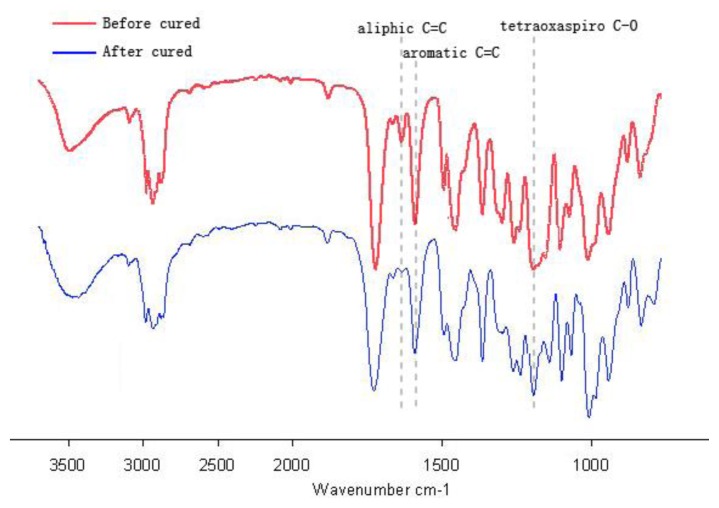
FTIR spectra exhibiting the reduction of aromatic C=C in peak height at 1635 cm^−1^ and tetraoxaspiro C–O absorbance at 1212 cm^−1^ associated with saturation of aliphatic C=C at 1590 cm^−1^ within the (BMSOC + BS)/T resin composite before and after cured.

Copolymerization reactions and the ring-opening reactions of the matrix resins were measured by FTIR. Figure 1 exhibits the reduction in peak height at 1635 cm^−1^ and tetraoxaspiro C–O absorbance at 1212 cm^−1^ associated with saturation of aliphatic C–C at 1590 cm^−1^ within the (BMSOC + BS)/T resin composite before and after cured. In comparison with the dental matrix resins before and after cured, aromatic C=C (1590 cm^−1^) peak almost kept invariable, while the aliphatic C=C (1635 cm^−1^) was obviously reduced, which meant that the number of the residual C=C (1635 cm^−1^) decreased, indicating the occurrence of the copolymerization reactions. Meanwhile, tetraoxaspiro C–O absorbance at 1212 cm^−1^ was significantly reduced, which means the occurrence of the ring-opening reactions.

**Figure 2.**
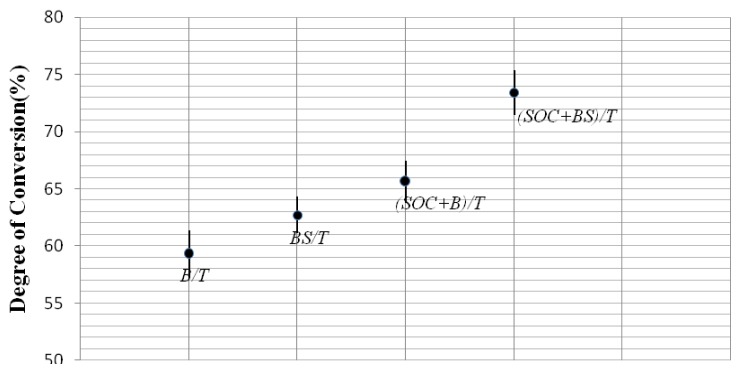
The degree of conversion of the four groups of resin composites.

The degree of conversion which was calculated from the equivalent aliphatic/aromatic molar ratio from polymerized and unpolymerized samples is presented in Figure 2. A one-way ANOVA and LSD test revealed significant differences between the DC of B/T and BS/T, (BMSOC + B)/T and (BMSOC + BS)/T, B/T and (BMSOC + B)/T, BS/T and (BMSOC + BS)/T (*p* < 0.05). The DC of (BMSOC + BS)/T tested after cured 24 h was significantly increased compared with the conventional dental matrix resins Bis-GMA/TEGDMA (B/T) (*p* < 0.05). The results suggest that BisS-GMA is more effective than Bis-GMA when referring to the degree of conversion, which is consistent with prior work. The explanation of superiority of BisS-GMA originates from the different structure between the two monomers. The oxygenic atom of O=S=O bond in BisS-GMA has a stronger electronegative power than methyl when connected with tertiary carbon in Bis-GMA, so the rate of reaction is speeded up and the degree of conversion is increased. Additionally, the presence of vinyl functionality in the BMSOC increased the reactivity with the bifunctional BisS-GMA. Degree of conversion is directly related to polymerization shrinkage stress [21]. In addition, the clinical performance of composites including mechanical properties, chemical stability and longevity of the composite depends on degree of conversion of the monomers in the material. Residual of monomers is an inevitable problem. The degree of conversion never achieves 100% and monomers that have not reacted remain within the polymer network, which damage the mechanical qualities of the restoration and are potentially toxic to surrounding tissues [22]. Higher degree of conversion is crucial to better mechanical properties, chemical stability and longevity of the composite [23]. The novel dental photocurable resin composite based on BisS-GMA as a resin matrix monomer and the expanding monomer BMSOC has a higher degree of conversion, which can indicate potentially valuable mechanical qualities.

### 2.2. Wettability Properties

Measurement of the contact angle at the solid-air-liquid meeting point is a widely known technique used to investigate wettability of solid substrates. The means and standard deviations of the contact angles of the studied groups are summerized in Table 1. The contact angle was significantly different between any two groups of composites except BS/T and (SOC + B)/T. The contact angle of the (SOC + BS)/T composite was significantly higher than that of (SOC + B)/T (*p* < 0.05). The addition of SOC increased significantly the contact angle (*p* < 0.05). Gan *et al.* reported that the contact angle data of the commercially available resin-based composites ranged between 31.5° and 64.5° under distilled water [24]. The contact angle of the novel composites with 58.9° is within this range and has a relatively higher value, which means lower wettability. Lower wettability of the composite resin surface and less water sorption contribute to the reduction of staining, and is an effective factor affecting plaque accumulation [25]. Secondary caries are the main reason for restoration replacement in operative dentistry. Therefore, dental materials with low wettability properties are preferable to limit the adhesion and proliferation of pathogens and, consequently, to prevent secondary caries and improved ability to limit the adhesion and proliferation of pathogens.

### 2.3. Volumetric Shrinkage, Contraction Stress and Compressive Strength

**Figure 3.**
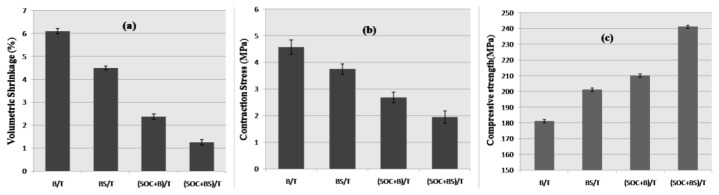
Volumetric shrinkage (**a**); contraction stress (**b**) and compressive strength (**c**) of the four groups of resin composites.

Figure 3 presents a summary of the volumetric polymerization-shrinkage, contraction stress and compressive strength of the four groups of composite resins investigated in this study. Values for BisS-GMA-containing test materials exhibited statistically significant (*p* < 0.05) lower shrinkage values in comparison to the corresponding Bis-GMA-containing resins. Values for BMSOC-containing test materials exhibited statistically significant (*p* < 0.05) lower shrinkage values than the corresponding BMSOC-free resins. The contraction stress of the resin composites corresponded to the result of shrinkage. (SOC + BS)/T resins exhibited the lowest shrinkage and contraction stress (*p* < 0.05).

CS testing is considered to be one of the most reliable methods to determine the mechanical strength of dental composites. The mechanical properties of CS of the resin composites containing 70 wt % of inorganic filler were examined. The values reported in MPa were the average number from the measurement of 12 replicate specimens, and computer generated automatically. It was observed that the groups of (SOC + B)/T and (SOC + BS)/T composites had significantly increased with respect to B/T and BS/T composites respectively. In addition, the CS of groups containing BisS-GMA had a higher value than Bis-GMA-containing composites. The CS was significantly different between any two groups of composites except BS/T and (SOC + B)/T (*p* > 0.05).

Polymerization shrinkage and subsequent contraction stresses contribute to various clinical challenges such as reduced marginal integrity, poor mechanical properties and even cracks in healthy tooth structure [26]. Comparison of the volumetric shrinkage results reported in this study, with recently published volumetric shrinkage data of commercially available resin-based composites, indicates that the novel composites shrank less than the great majority of commercially available composites whose reported volumetric shrinkage ranged between 1.5% and 3.5% [27,28]. BMSOC belongs to a class of expanding monomers [29,30], which are reported to produce a volumetric expansion during ring-opening polymerization. However, it is unlikely that SOC monomers could be used alone to produce dental composites, because they display poor curing characteristics [31]. An alternative approach would involve blending an SOC monomer with other monomers to achieve a system in which the amount of volumetric shrinkage caused by polymerization is minimized. This study, therefore, sought to decrease volumetric shrinkage of the resin composites. A BisS-GMA/TEGDMA mixture with BMSOC was used. BisS-GMA has a larger molecular weight, indicating that this monomer has a greater hydrodynamic radius than Bis-GMA according to the Stokes-Einstein equation, which would contribute to the observed lower volumetric shrinkage. A very significant reduction in volumetric shrinkage and contraction stress was observed for all SOC-based composites as compared to SOC-free. In contrast to the polymerization reaction of the methacrylates, the ring-opening polymerization of the BMSOC expanding monomer occurs via the cleavage and opening of ring-structures that gain apace and counteract the inevitable loss of volume. (SOC + BS)/T resin composites presented the lowest polymerization shrinkage due to the cationic ring-opening polymerization of the expanding monomer, which can explain the low polymerization stress.

Comparison of the compressive strength results reported in this study with recently published data of commercially available resin-based composites [32] indicates that the novel composites can meet clinical application. The novel composites had better mechanical property. There may be two explanations as follows. First, the effect of volumetric shrinkage was the most important and interrelated, which can directly influence the shrinkage stresses. Stresses that develop in bonded light-curable dental composite are closely related to the mechanical properties. Second, in the crosslinking polymerization system, degree of conversion determined the mechanical properties strongly.

## 3. Experimental Section

### 3.1. Synthesis and Characterization of BMSOC

**Scheme 1.**
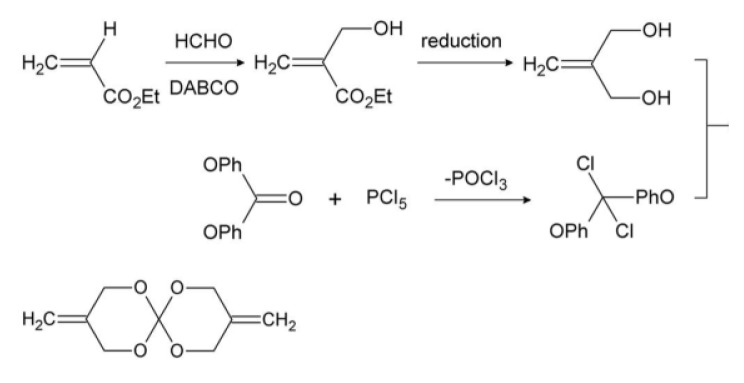
The main synthetic steps of BMSOC.

3,9-Dimethylene-1,3,5,7-tetraoxa-spiro[5,5]undecane (BMSOC), through synthesizing 2-methylene-1,3-propanediol which is derived from ethyl acrylate and formaldehyde and 1,1-dichloro-1,l-diphenoxymethane which is derived from diphenyl carbonate and phosphorous pentachloride on the basis of multistep reactions, was obtained. The main synthetic steps of BMSOC were shown in Scheme 1.

**Figure 4.**
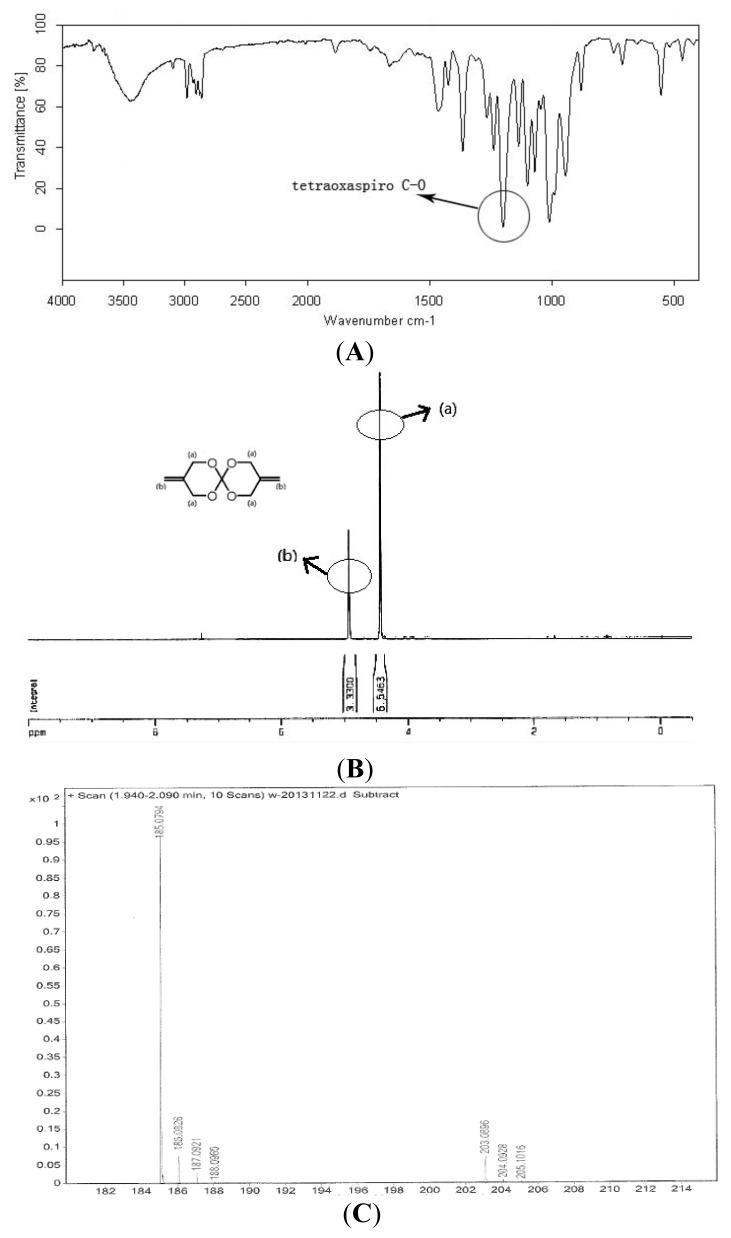
FTIR, ^1^H NMR and MS of BMSOC. (**A**) FTIR spectra of BMSOC; (**B**) ^1^H NMR spectra of BMSOC; (**C**) MS of BMSOC.

The target product was white solid and characterized by FTIR, ^1^H NMR and MS analyses (Figure 4). Yield: 86%. GC: 95%. ESI-HRMS [M + H]^+^ calculated for: 184.0736, found C_9_H_12_O_4_: 184, found 185.0794. IR (KBr): 3097 cm^−1^ (C=C), 1212 cm^−1^ (tetraoxaspiro C–O). ^1^H NMR (300 MHz, CDCl_3_) δ: 4.44 (m, 8H), 4.93 (m, 4H). Melting point: 79.8–81.2 °C. The result is consistent with that previously reported [33].

### 3.2. Materials and Preparation of Test Formulation

**Figure 5.**
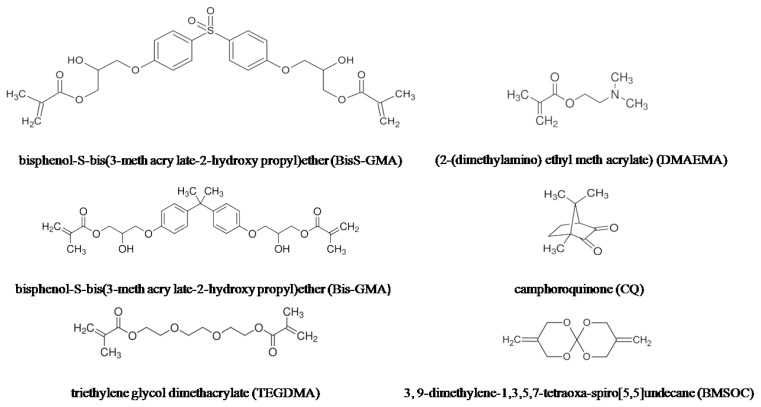
The chemical structure of materials used in the four groups of composites.

BisS-GMA was synthesized at Chengdu institute of organic chemistry, the University of the Chinese Academy of Sciences. The molecular structure of BisS-GMA was finally confirmed by FTIR and ^1^H NMR according our previously study [20]. Conventional dental matrix resins Bis-GMA, TEGDMA, the photoinitiator system consisting of camphoroquinone (CQ) and (2-(dimethylamino) ethyl meth acrylate) (DMAEMA) and inorganic fillers SiO_2_ were obtained from Sigma-Aldrich Co., Milwaukee, WI, USA. The particle size and shape of the filler SiO_2_ were larger than 230 mesh. Before being mixed in matrix resin, the SiO_2_ micro powder was modified by the coupling agent KH-570 [34] (Nanjing Crompton Shuguang Organosilicon Co., Nanjing, China). Briefly, a mixture of xylene and SiO_2_ (30 mL:1 g) was ultrasonically dispersed, and then the modifying agent (KH-570) was added dropwise into the dispersion under continuous agitation. The mixture was stirred for 8 h in the boiling state to obtain the modified SiO_2_ which could be mixed up with the organic matrix resin. The chemical structure of materials used in the composites is depicted in Figure 5.

### 3.3. Preparation of Test Formulation

Four groups of dental matrices with TEGDMA (30 wt %) which contained Bis-GMA/BisS-GMA and/or BMSOC were prepared as follows: Bis-GMA, BisS-GMA, BMSOC/Bis-GMA (3:7), BMSOC/BisS-GMA (3:7), with 1 wt % CQ as an initiator and 2 wt % DMAEMA as an accelerator. Mixing with inorganic fillers SiO_2_ of 70 wt %, the corresponding to the four groups of dental composites were prepared: B/T, BS/T, (SOC + B)/T and (SOC + BS)/T. Composition of each group has been summarized in Table 2.

### 3.4. Wettability Measurement

The wettability of the samples was assessed by measuring the contact angles of distilled water on composite resins with a contact angle measuring system (Sessile Drop, DSA30, IL4200, Hamburg, Germany). The contact angle was defined as the angle at which the liquid interface met the solid surface of the composite disc at four points on each sample, and the mean of the points was reported as the contact angle of each sample. The surface of the drop was monitored constantly, and the contact angle was measured just after 20 s, when the droplet was stabilized [35].

### 3.5. Degree of Conversion

Degree of conversion (DC) was measured by Fourier Transformation Infrared-FTIR [36] (Prestige21; Shimadzu, Columbia, ML, USA). Using a metallic mold of 4.5 mm diameter, 1 mm thick, specimens were prepared with each of the photoactivation protocols. A curing light (3M-ESPE, St Paul, MN, USA) at 650 mW/cm^2^ was used. The uncured and cured samples of the composite were placed above a horizontal zinc crystal. Measurements were made 24 h after photo activation (*n* = 5). Absorption spectra were recorded in a transmission mode using 16 scans at a resolution of 4 cm^−1^. The ratio of maximum absorption was determined using the base line method considering the peaks of 1635 cm^−1^ for aliphatic, and 1590 cm^−1^ for aromatic, chains. The degree of conversion was calculated from the equivalent aliphatic/aromatic molar ratio from polymerized (*P*) and unpolymerized (*U*) samples using the formula:

DC (%)=(1-P/U)×100

### 3.6. Volumetric Shrinkage

The VS values of the composite specimen were measured using a video-imaging device [37] (AccuVol; Bisco Inc., Schaumburg, IL, USA) in the single-view mode at room temperature (23–24 °C). Each specimen (*n* = 7) was shaped into a semisphere (with volumes averaging approximately 7 μL) and placed on a polytetrafluoroethylene pedestal in front of a CCD camera within the device. Special software was employed for the acquisition and processing of images. The uncured sample was allowed to rest for 1 min to eliminate the influence of slumping on the measurement and was later exposed to 40 s of light curing, with the curing unit tip positioned 1–2 mm from the specimen. The volume shrinkage image was recorded five min after light-curing in order to lower the temperature to room temperature.

### 3.7. Polymerization Contraction Stress

The polymerization-induced contraction stress was determined with a universal testing machine (EZ-Test, Shimadzu, Japan) with a fixture holding the composite material on a glass plate parallel to the flat surface of the steel cylindrically shaped rod with a six mm diameter [38,39]. A steel rod was fixed in the specimen-holder and connected to the crosshead with the load-cell of the universal testing machine. A sufficient amount of resin was applied to the glass surface and the cross-head was lowered towards the glass plate until the distance between the steel specimen surface and the glass surface was 2 mm. Excess resin was removed while shaping the specimen to a cylindrical form (diameter = 6 mm, height = 2 mm). The composite sample (*n* = 7) was light-cured for 40 s. The axial contraction stress development was recorded continuously from the start of curing until 30 min had elapsed.

All measurements above are presented as mean ± standard deviation and compared with the one-way ANOVA tests and LSD tests using SPSS (Chicago, IL, USA) 17.0 software. A value of *p* < 0.05 was considered statistically significant.

### 3.8. Compressive Strength (CS) Measurement

Bar-shaped specimens (*n* = 12) were prepared from each experimental material using copper cylindrical molds with dimensions of 6 mm (in diameter) × 4 mm (in height). The composite was cured for 40 s in three consecutive and overlapping sections to ensure the most complete polymerization possible. The specimens were stored in distilled water in an incubator (Thermal Fisher Scientific, Waltham, MA, USA) at 37 °C for seven days and then were subjected to compressive testing using the Universal Testing Machine (Instron 5800; Instron Corp., Norwood, MA, USA) with a load cell of 50 N at a crosshead speed of 1 mm/min, in accordance with ADA specification No. 27. The results were recorded in megapascals (MPa).

## 4. Conclusions

The aim of this study was to investigate the modification of Bis-GMA/TEGDMA dental resin composites with unsaturated bismethylene spiroorthocarbonate expanding monomer and BisS-GMA matrix monomer. The degree of conversion, volumetric shrinkage, contraction stress and microleakage properties were studied. The results indicated that the addition of unsaturated bismethylene spiroorthocarbonate expanding monomer and BisS-GMA matrix monomer mixtures reduced the polymerization-shrinkage and contraction stress in comparison with conventional methacrylate-based composites. Meanwhile, the increased degree of conversion, higher contact angle and improved mechanical properties of the novel resin composites were acquired, which make these new types of resin composites worthy of further study. Ongoing investigations into their physicochemical properties, biocompatibility and the ring-opening mechanism involved, are currently in progress and will be reported in due course.

## Figures and Tables

**Table 1.** The contact angles of the composites (mean ± sd).

Composites	Contact angle (°)
B/T	45.3 (3.2) ^a^
BS/T	51.6 (2.0) ^b^
(SOC + B)/T	52.7 (2.5) ^b^
(SOC + BS)/T	58.9 (3.0) ^c^

The different letters (a–c) indicate statistically significant differences between the composites (*p* < 0.05); The same letter denotes values that are not significantly different. (*p* > 0.05).

**Table 2.** Materials used in this study.

Resin composites (ratio)	Composition (abbreviation)	Photoinitiator system content(weight)
B/T (3:7)	Bis-GMA (B); TEGDMA (T)	CQ (1%)/DMAEMA (2%)
BS/T (3:7)	BisS-GMA (BS); TEGDMA (T)	CQ (1%)/DMAEMA (2%)
a (SOC + B)/T (3:7)	BMSOC (SOC); Bis-GMA (B); TEGDMA (T)	CQ (1%)/DMAEMA (2%)
a (SOC + BS)/T (3:7)	BMSOC (SOC); BisS-GMA (BS); TEGDMA (T)	CQ (1%)/DMAEMA (2%)

a The proportion of SOC and B/BS is 30% to 70%. BMSOC: 3,9-dimethylene-1,3,5,7-tetraoxa-spiro[5,5]undecane; Bis-GMA: 2,2-bis[4(2-hydroxy-3-methacryloylpropyloxy)phenyl]propane; BisS-GMA: bisphenol-*S*-bis (3-methacrylato-2-hydroxy propyl) ether; TEGDMA: striethylene glycol dimethacrylate; CQ: camphorquinone; DMAEMA: 2-(dimethylamino) ethyl methacrylate.
